# Non-invasive Transdermal Delivery of Human Insulin Using Ionic Liquids: *In vitro* Studies

**DOI:** 10.3389/fphar.2020.00243

**Published:** 2020-04-23

**Authors:** Ludmilla R. Jorge, Liliam K. Harada, Erica C. Silva, Welida F. Campos, Fernanda C. Moreli, Gustavo Shimamoto, Jorge F. B. Pereira, José M. Oliveira, Matthieu Tubino, Marta M. D. C. Vila, Victor M. Balcão

**Affiliations:** ^1^PhageLab – Laboratory of Biofilms and Bacteriophages of University of Sorocaba, Sorocaba, Brazil; ^2^Institute of Chemistry, University of Campinas, Campinas, Brazil; ^3^CIEPQPF-Department of Chemical Engineering, University of Coimbra, Coimbra, Portugal; ^4^Department of Biology and CESAM, University of Aveiro, Aveiro, Portugal

**Keywords:** human insulin, ionic liquids, choline geranate, choline oleate, transdermal permeation, chemical and biological characterization

## Abstract

In this research project, synthesis and characterization of ionic liquids and their subsequent utilization as facilitators of transdermal delivery of human insulin was pursued. Choline geranate and choline oleate ionic liquids (and their deep eutectic solvents) were produced and characterized by nuclear magnetic resonance (^1^H NMR), water content, oxidative stability, cytotoxicity and genotoxicity assays, and ability to promote transdermal protein permeation. The results gathered clearly suggest that all ionic liquids were able to promote/facilitate transdermal permeation of insulin, although to various extents. In particular, choline geranate 1:2 combined with its virtually nil cyto- and geno-toxicity was chosen to be incorporated in a biopolymeric formulation making it a suitable facilitator aiming at transdermal delivery of insulin.

## Introduction

Insulin therapy is used in the treatment of *Diabetes Mellitus*, controlling hyperglycemia in patients suffering from type-1 diabetes. *Diabetes Mellitus* is a metabolic disorder characterized by hyperglycemia, glycosuria, hyperlipidemia, negative nitrogen balance, and sometimes ketonemia. Insulin delivery systems that are currently available for the administration of insulin include syringes, insulin infusion pumps, jet injectors, and pens (Panchal et al., 2011). However, a major drawback of the current forms of insulin therapy lies in their invasive nature. This has prompted the development of an artificial pancreas with a closed-loop system, and exploration of newer insulin delivery methods such as transdermal, buccal, oral, pulmonary, nasal, ocular and rectal routes (Mamatha et al., 2015). Since the skin is the largest organ in the human body, the transdermal route becomes an appealing route for insulin delivery (Dragicevic and Maibach, 2015; Jung and Maibach, 2015). Its main advantages are its complacency and the possibility of controlled release over time, elimination of the possibility of drug degradation in the gastrointestinal tract and also painless administration (Khafagy et al., 2007). However, skin composition makes this organ an effective protective barrier, being impermeable to foreign molecules, especially large and hydrophilic molecules such as insulin. The stratum corneum, the outermost layer of the skin, is responsible for this impermeability, being constituted by an association between keratinocytes and lipids (Lassmann-Vague and Raccah, 2006; Khafagy et al., 2007). In order to overcome this obstacle, and to allow insulin absorption, various methods of weakening the stratum corneum have been tested over time, namely methods including absorption promoters, iontophoresis, sonophoresis, and micro-needles (Lassmann-Vague and Raccah, 2006; Khafagy et al., 2007). Absorption promoters may increase cutaneous permeability by increasing the partition coefficient of the administered drug or the thermodynamic action thereof, and by modifying the composition of the stratum corneum by breaking the lipid structure (Lassmann-Vague and Raccah, 2006; Li et al., 2008). In their work, Zakrewsky et al. (2014) reported a polyvalent ionic liquid, choline geranate, exhibiting minimal toxicity to both epithelial cells and skin, and effective improvement of transdermal permeation to drug delivery. The biological effects of ionic liquids, including transdermal delivery and virtual absence of cytotoxicity, are fully related to the chemical properties of ionic liquids and their constituent cations and anions (Zakrewsky et al., 2014). Ionic liquids are able to solubilize amphipathic molecules and, therefore, act as ideal solvents for topical and transdermal delivery of drugs. The ionic liquid molecules are likely to slip through the fatty compounds that make up the skin cells, creating small transient openings through which bioactive molecules (carried by ionic liquid) can permeate. Variation in human skin permeability has been reported even among individuals (both inter- and intra-individual variations), and hence pig skin is frequently used as an alternative to human skin in percutaneous absorption studies aiming at developing transdermal formulations (Todo, 2017).

In this sense, the aim of the research work that has been undertaken was the development and characterization of biocompatible ionic liquids (ILs) for integration in a biopolymeric film with insulin, aiming at its transdermal delivery, using porcine skin as skin model.

## Materials and Methods

### Materials

#### Chemicals

All reagents used were of analytical grade and were used without further purification. Tap water was purified in a Master System All (model MS2000, Gehaka, São Paulo, Brazil) to a final resistivity of ca. 18.18 MΩ.cm and conductivity of 0.05 μS cm^–1^. Human insulin (ref. Novolin^®^ N, 100 UI/mL, [protein] = 2.395 mg/mL, register MS No. 1.1766.0004.002-1; recombinant human insulin isophane suspension that is structurally identical to human insulin) was purchased from Novo Nordisk A/S (Kalundborg, Denmark). Coomassie Brilliant Blue G250, ortho-phosphoric acid (85%, v/v), UVASOL^TM^ ethanol for spectroscopy (99.8%, v/v), Geranic acid (85% stabilized) and Choline bicarbonate were purchased from Sigma-Aldrich (St. Louis MO, United States). Oleic acid (P.A.) was purchased from LABSYNTH – Produtos para Laboratório (Diadema, Brazil). Glycerol was purchased from Cinética (Jandira, Brazil). HPLC-grade methanol (LiChrosolv^®^, CAS-No: 67-56-1) was purchased from Merck (Darmstadt, Germany). Xanthan (XANTURAL^®^ 180) gum (CAS No. 11138-66-2; MW of 241.11 g/mol) was a kind gift from CPKelco (Limeira, Brazil). Methylparaben was acquired from Synth (Diadema, Brazil).

#### Biological Materials

The 3T3 and HaCaT (immortalized human keratinocytes) cell lines used in the genotoxicity (Comet^TM^) and MTT assays were purchased from Sigma-Aldrich (St. Louis MO, United States). The cells were maintained at 37°C under moist atmosphere with 5% CO_2_, in Dulbecco’s modified Eagle’s medium (DMEM) containing D-glucose (4.5 g/L), L-glutamine (584.0 mg/L), sodium pyruvate (100 mg/mL), and sodium bicarbonate (3.7 g/L) (Gibco Life Technologies, São Paulo, Brazil), supplemented with 10% (w/w) fetal bovine serum and 1% (w/w) antibiotics [penicillin (100 IU/mL) and streptomycin sulfate (100 μL/mL)].

### Experimental Procedures

#### Synthesis of Ionic Liquids (IL) and Their Deep Eutectic (DES) Solvents

The IL and DES were prepared by neutralizing to geranic acid (GA, donor in the hydrogen bond) or oleic acid (OA, donor in the hydrogen bond) with choline bicarbonate (CB, cation) according Zakrewsky et al. (2014). To prepared the IL was employed a ratio of choline bicarbonate (CB, cation) to GA or OA of 1:1 obtained choline geranate ionic liquid (CG 1:1) or choline oleate ionic liquid (CO 1:1). To prepared the DES a ratio of CB to GA or OA of 1:2 was used obtained choline geranate deep eutectic solvent (CG 1:2) or choline oleate deep eutectic solvent (CO 1:2).

#### Physicochemical Characterization of Ionic Liquids

##### Water content determination via Karl-Fischer titration

The moisture contents of the ionic liquids were determined via a biamperometric titration of previously weighed amounts injected into a Karl-Fischer’s reaction vessel (684 KF Coulometer from Metrohm, Herisau, Switzerland).

##### Nuclear magnetic resonance (^1^H NMR) analyses

All of the ^1^H NMR spectra were recorded in a Bruker Avance III 500 MHz NMR spectrometer (Bruker, Billerica, MA, United States). To obtain the spectra, a sample of ∼20 μL of each sample was dissolved in 600 μL of CDCl_3_, containing tetramethylsilane (TMS) as an internal reference (0.00 ppm), using the following experimental conditions: pulse program, zg30; spectral width, from −4.00 to 16.00 ppm; spectral size, 32768 points; 90° pulse, 11.75 μs; delay, 1 s; and number of scans, 16.

##### Oxidative stability

The oxidative stability of IL samples (CG 1:1, CG 1:2, CO 1:1, and CO 1:2) was determined in a Biodiesel Rancimat from Metrohm AG (model 873, Herisau, Switzerland). Initially, 2 g of each IL were placed into Rancimat standard tubes and subjected to the induced oxidation conditions with heating at 110°C and an air flow of 10 L h^–1^. During the test, the conductivity of the cell containing 60 mL of water was monitored until it reached 20 μS cm^–1^ of conductivity. In order to obtain the estimated oxidative stability at any temperature, forced oxidation was performed at different temperatures (viz. 55, 75, 90, and 110°C). Calibration curves were produced by plotting Ln (t, h) = *f*{T,°C}.

#### Biological Characterization of Ionic Liquids

##### Cytotoxicity potential of plain ionic liquids agar disk-diffusion assay

The cytotoxicity potential of plain ILs and their respective DES was evaluated via the agar disk-diffusion methodology using cell lineage 3T3, according to the procedure described by Rocha et al. (2017). The readings of the inoculated plaques were made macroscopically, where the presence of cytotoxicity was observed by the formation of a clear halo around the toxic material corresponding to the dead cells, and microscopically, for the morphological changes of the cells surrounding the sample.

##### Mitochondrial activity (MTT) assay

Evaluation of the cytotoxicity potential of plain ILs to HaCaT (immortalized human keratinocytes) cell lines (assessment of cellular viability) was also carried out using the MTT (3-(4,5-dimethylthiazol-2-yl)-2,5-diphenyltetrazolium bromide) assay, according to the procedure described by Rocha et al. (2017).

##### Evaluation of the DNA damage potential (genotoxicity) of plain ionic liquids, via the Comet^TM^ assay

3T3 cells were placed in contact with the samples (CG 1:1, CG 1:2, CO 1:1, and CO 1:2) (for CO 1:1 and CO 1:2, 2.5 g IL were diluted in 250 mL of ultrapure water and, from the resulting solution, 300 μL were used for the test; for CG 1:1 and CG 1:2, 250 μL of IL were diluted in 10 mL of ultrapure water and, from the resulting solution, 300 μL were used for the test), with the negative being plain DMEM medium in contact with the 3T3 cells) during a period of 1 h. Tests were carried out in triplicate for each group, following the procedure described in detail by Rocha et al. (2017).

#### Synthesis of Biopolymeric Films Integrating Ionic Liquids and Human Insulin

##### Experimental factorial design for optimization of plain biopolymeric films with appropriate mechanical properties

A 3^2^ full factorial design approach (two variables each one set at three levels), thus requiring a total of nine formulations producing nine experiments, was applied to fully maximize the experimental efficiency with a minimum of experiments. The two different variables (xanthan gum and bacterial nanocellulose concentrations) at three levels each, low (−1), medium/central (0) and high (+ 1), and their influence upon the mechanical properties of the biopolymeric films produced were duly studied. The independent variables were xanthan gum and bacterial nanocellulose concentration, whereas the established dependent variables under scrutiny were the mean mechanical properties, viz. resistance to traction, resilience, and relaxation. For each independent variable, the low, medium and high values of the lower, central and upper levels were assigned a (−1), (0) and (+ 1) sign, respectively (Table 1).

**TABLE 1:** Full 3^2^ factorial design, providing the lower (−1), central (0) and upper (+1) level values for each variable; composition of the plain biofilms (base: 10 g) for optimization of the appropriate mechanical properties; and composition of the (optimized) biofilms integrating ionic liquid and human insulin.

Independent variables	Levels		
	Low level	Central level	Upper level
	(−1)	(0)	(+ 1)
Bacterial nanocellulose (%, w/w)	0.050	0.100	0.150
Xanthan gum (%, w/w)	0.500	1.000	1.500

Levels/mass				PVA (g)	Glycerol (g)	Methylparaben (g)	Ultrapure water (g)
Bacterial nanocellulose (g)		Xanthan gum (g)					
(−1)	0.005	(−1)	0.050	0.125	0.250	0.010	9.860
(−1)	0.010	(0)	0.050	0.125	0.250	0.010	9.550
(−1)	0.015	(+ 1)	0.050	0.125	0.250	0.010	9.550
(0)	0.005	(−1)	0.100	0.125	0.250	0.010	9.510
(0)	0.010	(0)	0.100	0.125	0.250	0.010	9.500
(0)	0.015	(+ 1)	0.100	0.125	0.250	0.010	9.500
(+ 1)	0.005	(−1)	0.150	0.125	0.250	0.010	9.460
(+ 1)	0.010	(0)	0.150	0.125	0.250	0.010	9.450
(+ 1)	0.015	(+ 1)	0.150	0.125	0.250	0.010	9.450

Biofilm #	Bacterial nanocellulose (g)	Xanthan gum (g)	PVA (g)	Glycerol (g)	Methylparaben (g)	Ionic liquid (CG1:2, mL)	Insulin NOVOLIN (mL)	Ultrapure water (g)
Biofilm 1	0.015	0.150	0.125	0.250	0.010	0.250	1.500	7.700
Biofilm 1	0.015	0.150	0.125	0.250	0.010	0.000	1.500	7.950

All formulations were produced according to the experimental factorial design depicted in Table 1, integrating constant amounts of PVA (1.25%, w/w; to induce polymerization), glycerol (2.50%, w/w; to induce plasticity) and methylparaben (0.1%, w/w; to inhibit fungal growth), thus yielding nine (plain) biopolymeric film formulations (Table 1).

##### Production of biopolymeric films integrating ionic liquid and insulin, departing from the optimum formulation arising from the experimental factorial design

After determination of the optimal biopolysaccharide levels leading to a suitable film in terms of mechanical properties (viz. adequate resistance to traction, relaxation and resilience) while maintaining a good level of malleability, such levels were used for production of a biofilm integrating human insulin and ionic liquid. Hence, a total of two biofilms were produced integrating sericin and ionic liquid (or not) according to the data included in Table 1.

#### Physicochemical and Biological Characterization of Biopolymeric Films Integrating Ionic Liquids and Human Insulin

##### Thermal analyses via differential scanning calorimetry (DSC)

Thermal characterization of the biopolymeric film integrating insulin and ionic liquid was accomplished via DSC. The DSC analyses were carried out using a differential scanning microcalorimeter also from TA Instruments (model MDSC 2910).

##### Fourier transform infrared spectrophotometry (FTIR) analyses

The FTIR spectrum of the biopolymeric film integrating insulin and ionic liquid was gathered using a Fourier Transform Infrared 100 Spectrophotometer from Agilent (model Cary 630, Santa Clara, CA, United States), in the wavenumber range from 4000 to 400 cm^–1^, with a resolution of 2 cm^–1^, and using Happ-Genzel apodization.

##### Mechanical resistance properties

Mechanical properties of the biopolymeric films were evaluated using a texturometer from Stabile Micro Systems (model TA-TX Plus, Godalming, United Kingdom), evaluating parameters such as resistance to traction, relaxation and resillience. The determination parameters were set as distance of 5 mm for the perforation resistance tests, distance of 2 mm for both resillience and relaxation tests, and a maximum force of 5 kg for all tests. All determinations were performed in triplicate. Sample dimensions were 2 cm × 5 cm for resistance to traction, and 2 cm × 2 cm for relaxation and resilience.

##### Cytotoxicity potential of plain biofilm and biofilm integrating choline geranate and human insulin agar disk-diffusion assay

The cytotoxicity potential of plain biofilm and biofilm integrating insulin and ionic liquid was evaluated via the agar disk-diffusion methodology, as described before, using cell lineage HaCat.

##### Tomographic analyses via X-ray transmission (XRT)

The tomographic images of the biofilm integrating insulin and ionic liquid were gathered using a 3rd generation computed X-ray transmission tomograph (Oliveira Junior and Martins, 2009) from Bruker microCT (model SkyScan 1174, Kontich, Belgium), following the procedure described by Rocha et al. (2017).

##### Dispersive energy scanning electron microscopy analyses (DESEM)

The surface and morphology of the biofilm integrating insulin and ionic liquid were observed in a dispersive energy scanning electron microscope (DESEM) from LEO Electron Microscopy/Oxford (model Leo 440i, Cambridge, United Kingdom) equipped with a EDS detector (model 6070, Cambridge, United Kingdom). Samples of the biofilm were either cut or cryo-fractured and sputter-coated with a Au film (200 Å thickness) via cathodic pulverization on a carbon layer produced by evaporation in a metalizing device (Sputter Coater) from EMITECH (model K450, Kent, United Kingdom). Microphotographs were gathered using electron beams with acceleration speeds of 20 keV and electric current of 100 mA, via random scanning.

#### Ionic Liquid-Assisted Transdermal Permeation of Human Insulin

The non-invasive ionic liquid-assisted delivery of insulin was evaluated via transdermal permeation studies in a DHC-6T Transdermal System from Logan Instruments, Corp. (Somerset, NJ, United States). For these assays, thawed porcine ear skin (prepared according Salerno et al., 2010) disks (0.5 mm thick × 30 mm ϕ_*ext*_) were cut and clamped in place between the acceptor and donor chambers on top of the Franz diffusion cell support (possessing a central hole with 15 mm ϕ_*ext*_). On top of these porcine ear skin disks, either 100 μL ionic liquid and 100 μL plain insulin or a biofilm disk (30 mm ϕ_*ext*_) integrating ionic liquid and insulin was placed. To the bottom glass receptacle of the Franz diffusion cell, 8 mL of (degassed) phosphate buffer (pH 7.4) were injected until touching the lower surface of the porcine ear skin disk. At pre-determined time intervals (viz. 0, 15, 30, 45, 60, 75, 90, 120, 150, 180, 360, 540, 720, 840, 1410, and 1440 min), 2 mL-samples were withdrawn from the receiving fluid beneath the porcine ear skin disk, and 2-mL of fresh phosphate buffer (pH 7.4) were duly added so as to reset the volume. Each sample was then assayed for protein quantification via a modification of the Bradford method for protein quantification, as described in detail by Robyt and White (1990) and Balcão et al. (1996). The experiment was terminated when a plateau was reached (and maintained) in the permeated protein concentration. Aliquots of 500 μL of the supernatant samples were added to 4.5 mL of the working solution of Coomassie Brilliant Blue G-250 [prepared according to the procedure described elsewhere (Robyt and White, 1990; Balcão et al., 1996)], incubated at room temperature for 5 min and absorbance was measured at 595 nm using disposable plastic cuvettes (Kartell) in a UV-Vis Spectrophotometer from Agilent (model Cary 60 UV-Vis, Santa Clara, CA, United States). A calibration curve for protein was prepared using solutions of bovine serum albumin (BSA) in phosphate buffer (pH 7.4) at several concentrations in the range 0–1000 μg/mL: Abs_595*nm*_ = 1.6554 x C_*prot*_/(0.1922 + C_*prot*_) (*r* = 0.99529).

## Results and Discussion

Biopolymeric films are very interesting for pharmaceutical and biomedical applications due to their virtually zero toxicity, potential for prolonged release of bioactive entities, their impermeability to bacterial cells, elasticity, among other properties such as being fully biodegradable and virtually inert biopolymers with respect to possible cyto- and geno-toxicities (Rossi et al., 2010). Insulin is a polypeptide hormone with a fully known chemical structure, of a protein nature, responsible for the reduction of glycemia (Robert, 2002). When insulin production is deficient, glucose accumulates in the blood and urine, leading to the destruction of cells due to a lack of supply and consequent onset of the condition known as *Diabetes mellitus*. For patients in this condition, insulin is provided by injections, or insulin pumps. Thus, and due to all the inherent advantages of bio-inspired and biomimetic systems, of which biopolymeric matrices are but an example, the development of a flexible and elastic biopolymeric film integrating insulin and a transdermal permeation facilitator such as a ionic liquid was sought, aiming at the structural and functional stabilization of insulin for its release and transdermal delivery.

### Water Content Determination of the Ionic Liquids via Karl-Fischer Titration

The water content of CB was ca. (22.12 ± 0.12)% (w/w), of OA was ca. (0.55 ± 0.01)% (w/w), of GA was ca. (1.30 ± 0.06)% (w/w), and of the ionic liquids was ca. (10.32 ± 0.02)% (w/w) for CO (1:1), ca. (6.17 ± 0.15)% (w/w) for CO (1:2), ca. (7.76 ± 0.37)% (w/w) for CG (1:1), and ca. (2.24 ± 0.09)% (w/w) for CG (1:2).

### Nuclear Magnetic Resonance (^1^H NMR) Analyses of the Ionic Liquids

**FIGURE 1:**
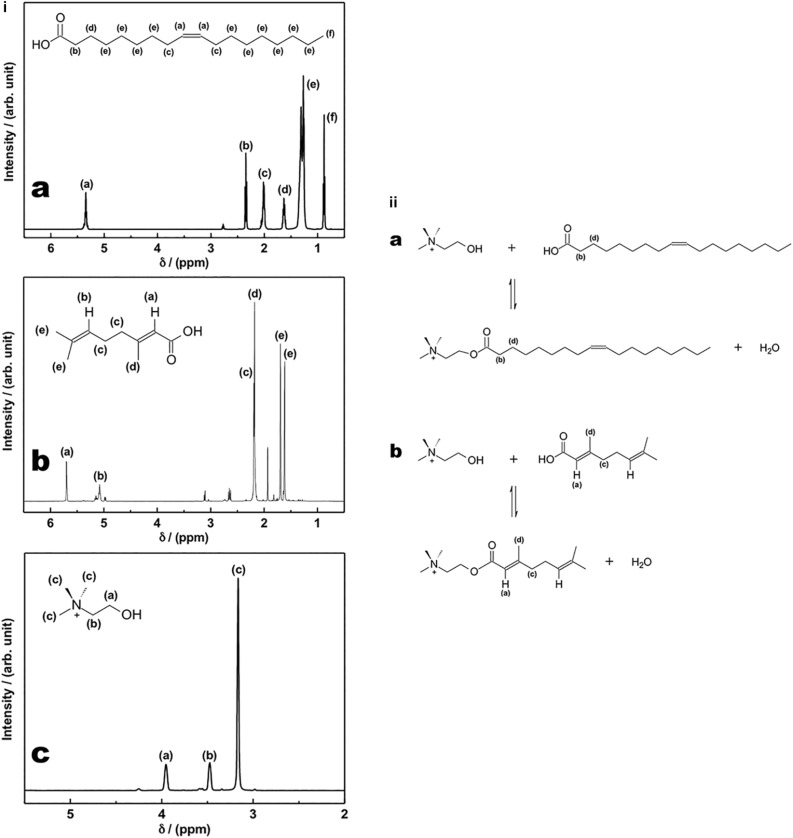
**(i)**^1^H NMR spectra of oleic acid **(a)**, geranic acid **(b)**, and choline bicarbonate **(c)**; **(ii)** Esterification reactions between **(a)** oleic acid and choline cation to produce choline oleate [hydrogens (b) and (d) are the most affected by NMR spectra, when the ^1^H NMR spectrum of the ester is compared with the acid one], and **(b)** geranic acid and choline cation to produce choline geranate [hydrogens (a), (c), and (d) are the most affected by NMR spectra, when the ^1^H NMR spectrum of the ester is compared with the acid one].

Figure 1i shows the assignment of ^1^H NMR signals for the starting materials: OA (Figure 1i-a), GA (Figure 1i-b), and CB (Figure 1i-c), respectively.

Figure 1ii-a shows the esterification reaction between OA and choline cation to produce CO. The transformation of the acid into an ester changes the chemical environment of the hydrogens near the carbonyl group. In Figure 1ii-a, the hydrogens (b) and (d), from OA, are the most affected by NMR spectra, when the ^1^H NMR spectrum of the ester is compared with the acid one.

**FIGURE 2:**
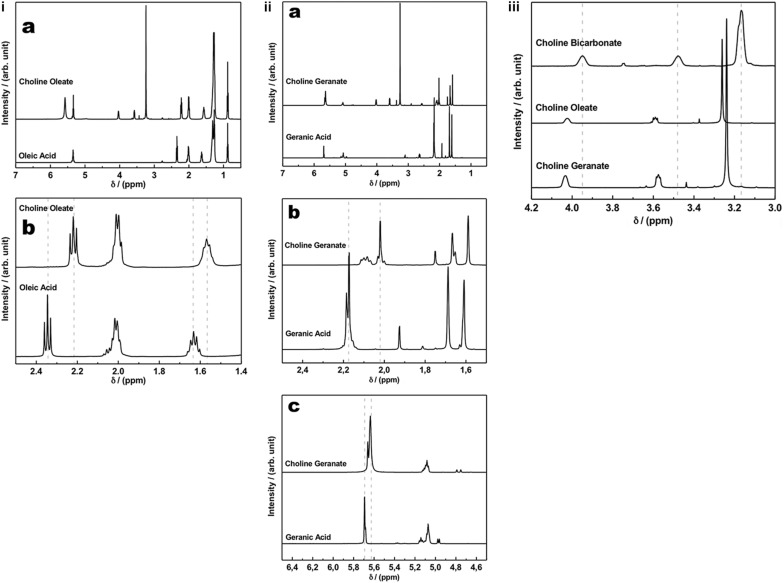
**(i)** ^1^H NMR spectra of choline oleate and of its starting material oleic acid **(a)**, together with an expansion to highlight the upfield shifts from 2.34 to 2.21 ppm and from 1.63 to 1.57, when these signals from the ester are compared with the respective signals from the acid **(b)**; **(ii)** ^1^H NMR spectra of choline geranate and its starting material, geranic acid **(a)** and two expansions, to highlight the upfield shifts from 2.18 to 2.02 ppm **(b)** and from 5.69 to 5.63 ppm **(c)**, when these signals from the ester are compared with the respective signals from the acid; **(iii)**^1^H NMR spectra of choline bicarbonate, choline oleate, and choline geranate, to highlight the downfield shifts when the signals from the ester are compared with the respective signals from the choline bicarbonate.

Figure 2i-a shows the ^1^H NMR spectra of CO and of its starting material, OA. In addition, Figure 2i-b shows an expansion to highlight the upfield shifts from 2.34 to 2.21 ppm, related to the alpha hydrogens to carbonyl, and from 1.63 to 1.57, related to the beta hydrogens to carbonyl, when these signals from the ester are compared with the respective signals from the acid.

Figure 1ii-b shows the esterification reaction between GA and choline cation to produce CG. The transformation of the acid into an ester changes the chemical environment of the hydrogens near the carbonyl group. In Figure 1ii-b, the hydrogens (a), (c), and (d), from GA, are the most affected by NMR spectra, when the ^1^H NMR spectrum of the ester is compared with the acid one. Figure 2ii-a shows ^1^H NMR spectra of CG and of its starting material, GA. Besides, Figures 2ii-b,c are two expansions to highlight the upfield shifts from 2.18 to 2.02 ppm, related to the hydrogens (c) and (d), and from 5.69 to 5.63 ppm, related to hydrogen (a), when these signals from the ester are compared with the respective signals from the acid. In Figure 2ii-b, it is also observed a better separation of the hydrogen signals (c) and (d) in the spectrum of the ester, when compared with the acid spectrum. The change in the hydrogen environment in the ionic liquid molecules will certainly have an effect on the permeability of the *stratum corneum*. A higher concentration in hydrogen ions not only limits the growth of pathogenic skin flora but also is required for the enzymatic lipid processing that results in an effective permeability barrier of the stratum corneum (Elias, 2005; Lee et al., 2006; Chan and Mauro, 2011).

In addition to the cited differences, in both Figures 2i,ii one can observe the signs of the choline cation structure. Figure 2ii shows the differences observed in the signals related to the choline cation structure in the CB, CO, and CG spectra.

### Cytotoxicity Potential of Plain Ionic Liquids

#### Agar Disk-Diffusion Assay

**FIGURE 3:**
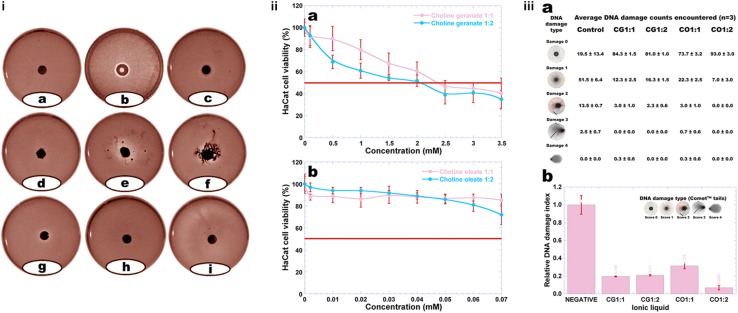
**(i)** Results from the cytotoxicity analysis of plain ionic liquids and their respective deep eutectic solvents via the agar disk-diffusion methodology using the cell lineage 3T3: **(a)** negative control, **(b)** positive control, **(c)** choline geranate 1:1, **(d)** choline geranate 1:2, **(e)** choline oleate 1:2, **(f)** choline oleate 1:1, **(g)** oleic acid, **(h)** geranic acid, and **(i)** choline bicarbonate; **(ii)** Results from the cytotoxicity analysis of plain ionic liquids via the mitochondrial activity (MTT) assay, using the cell lineage HaCaT: **(a)** choline geranate, and **(b)** choline oleate; **(iii)** Average DNA damage counts encountered in the Comet^TM^ test performed to the ionic liquids using 3T3 cells **(a)**, and relative DNA damage indexes of 3T3 cell line following exposure of cells to the different ionic liquids for 24 h [means of six determinations with associated σ: **3T3** (control, 1.000 ± 0.104; choline geranate 1:1, 0.196 ± 0.005; choline geranate 1:2, 0.210 ± 0.008; choline oleate 1:1, 0.315 ± 0.033; choline oleate 1:2, 0.070 ± 0.024)] **(b)**. The inserted photos picture the Comet^TM^ tails produced by the different types of damaged cells encountered.

The cytotoxicity potential of plain ionic liquids and their respective deep eutectic solvents was evaluated via the agar disk-diffusion methodology using the cell lineage 3T3 (Figure 3i).

The results obtained showed that there was no cell death caused by contact with GA (Figure 3i-h), with the cells presenting integrity under microscopical analysis. On the contrary, contact with CG 1:1 (Figure 3i-c), CG 1:2 (Figure 3i-d), CO 1:2 (Figure 3i-e), CO 1:1 (Figure 3i-f), OA (Figure 3i-g), and CB (Figure 3i-i), mild cytotoxicity was observed, presented by the clearer halo around the material sample, with the samples containing CO being the most toxic (Figures 3i-e,f).

### Mitochondrial Activity (MTT) Assay

Evaluation of the cytotoxicity potential of plain ionic liquids to HaCaT (immortalized human keratinocytes) cell line (assessment of cellular viability) was also carried out using the MTT assay (Figure 3ii). The half maximal inhibitory concentration (IC_50_, a measure of the effectiveness of a substance in inhibiting a specific biological or biochemical function, which determines the concentration of product needed to kill 50% of the cells) was not attained for all ionic liquids assayed (red line in Figure 3ii). Hence, such quantitative measure indicates how much of a particular substance is needed to inhibit a given biological process by half.

The analyses carried out with CG showed a loss of cell viability for both CG 1:1 and CG 1:2 (Figure 3ii-a), with the IC_50_ being determined via the Probit curve (a type of regression where the dependent variable can take only two values, hence being a popular specification for an ordinal or a binary response model) and producing the values IC_50_ = 2.45 and 2.01 mM for CG 1:1 and CG 1:2, respectively (Figure 3ii-a). The results show that, after 24 h, cell viability following exposure of HaCaT cells to CO 1:1 and CO 1:2 was higher than 70% (Figure 3ii-b) and, in this way, at the concentrations tested it was not possible to determine the IC_50_ values for CO.

### Determination of the DNA Damage Potential of Plain Ionic Liquids, via Comet^TM^ Assay

The extent of any potential DNA damage (genotoxic effects) promoted by the ionic liquids synthesized, viz. CO1:1 and CO1:2, and CG1:1 and CG1:2 in 3T3 cell line was assessed by the Comet^TM^ assay, which detects DNA strand breaks following either direct damage induced by the samples or indirect damaging effects linked to the DNA repair process. The Comet^TM^ assay is able to quantitatively detect the DNA damage caused by alkylating or oxidizing and intercalating agents. The DNA damage indexes were calculated according to the sizes of the cell tails produced. The tails were scored in five levels (0, 1, 2, 3 and 4), where zero and four represent the lowest and the highest comet tail sizes respectively, with the DNA damage index (DI) being calculated as

DI={(0×∑cellswithscore 0)+

(1×∑cellswithscore 1)+

(2×∑cellswithscore 2)+(3×∑cellswithscore 3)+

(4×∑cellswithscore 4)}/totalnumberofcells

The slides were analyzed using a single-blind-review in order to minimize variability. To evaluate any possible genetic damage to the cells imparted by the CO1:1, CO1:2, CG1:1, and CG1:2 samples, the Comet^TM^ test was carried out with 3T3 cell line, allowing to observe significant differences between the control and the 3T3 cell line that was exposed to the CO1:1, CO1:2, CG1:1, and CG1:2 samples (Figure 3iii).

The statistical analysis performed to the data gathered from this test was carried out using GraphPad Prism v. 7.0 (GraphPad Software, Inc., La Jolla, CA, United States). Regarding the analysis performed to compare between samples (including the control), no statistical different results were found between samples, but the statistical difference between the samples and the negative control was significative (Figure 3iii-b). A statistically significant difference was obtained between the control and CO1:1, CO1:2, CG1:1 and CG1:2 samples, meaning that a possible (although not to a great extent) genotoxic effect may be attributed to the control but not to the ionic liquid samples. The high relative DNA damage index imparted by the control (Figure 3iii) was due to a high count of cells with types 1, 2, and 3 damages. No type 4 damages were found for the HUVEC cells treated with either the control and any of the ionic samples tested. All ionic liquid samples promoted an intermediate level of type 1 damage irrespective of the type of ionic liquid (Figure 3iii-a). Low levels of type 3 damages were produced by all ionic liquid samples. This means that the ionic liquid samples tested do not possess characteristics that promote lesions in the DNA to a great extent, as apparent from the absent cell counts with levels of type 3 damage (Figure 3iii-a). However, the Comet^TM^ evaluation is performed with a cellular exposition of 1 h, because it is a pre-testing procedure without the need of any cellular divisions. Figure 3iii-b represents the genotoxic effects (relative DNA damage index) of 3T3 cell line incubated with plain DMEM culture medium (negative) and with the CO1:1, CO1:2, CG1:1, and CG1:2 samples. For the 3T3 cells, the ionic liquid samples tested showed no significant genotoxic effects whatsoever. These results are in clear agreement with the very low IC_50_ values encountered for the ionic liquids (Figure 3ii). Hence, the lack of extense cytotoxic effects is in line with the lack of extense DNA damages, which in turn is in clear agreement with results published by Seabra et al. (2014).

### Oxidative Stability of Plain Ionic Liquids, via Rancimat^TM^ Assay

**FIGURE 4:**
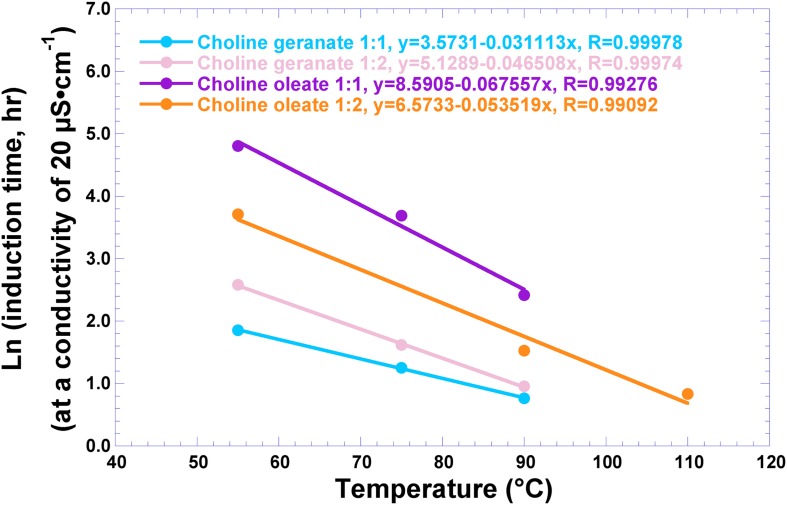
Oxidative stability of the several ionic liquids produced, via the Rancimat^TM^ assay at different temperatures (viz. 55, 75, 90, and 110°C).

The oxidative stability of ionic liquid (CO1:1, CO1:2, CG1:1, and CG1:2) samples was evaluated via forced oxidation at different temperatures (viz. 55, 75, 90, and 110°C) and fitting the data to a linear semilogarithm curve, viz. Ln (t, h) = *f*{T,°C} (Figure 4).

The thermo-oxidative stability of the ionic liquids (CO1:1, CO1:2, CG1:1, and CG1:2) was evaluated by the lipid oxidation induction times. Oxidative stability is an important parameter in the characterization of fats and oils. The Rancimat^TM^ method is based on the conductometric determination of volatile degradation products, and features automatic plotting of the conductivity against time (Laübli and Bruttel, 1986). The changes in chemical and physical properties such as viscosity, deposit formation and weight loss, depending of oxidation, may interfere with the quality of products containing ionic liquids in their formulation, and therefore researchers try to answer questions concerning the stability of ionic liquids (Siedlecka et al., 2011). In this sense, the lipid oxidation induction times of the synthesized ionics liquids were determined via the Rancimat^TM^ method with the objective of estimating their oxidative stability. However, it is known that the mechanisms of lipid oxidation under Rancimat^TM^ conditions and at ambient temperature are substantially different (Gharby et al., 2016). The calculated values of the oxidation induction times using the linear fittings depicted in Figure 4, at 40°C, were: 10.26 h (CG 1:1), 23.64 h (CG 1:2), 311.76 h (CO 1:1), and 84.14 h (CO 1:2). Hence, it can be concluded that the synthesized ionic liquids are stable for use in biomedical products.

### Statistical Optimization of Biopolymeric Films Integrating Ionic Liquid and Human Insulin

**FIGURE 5:**
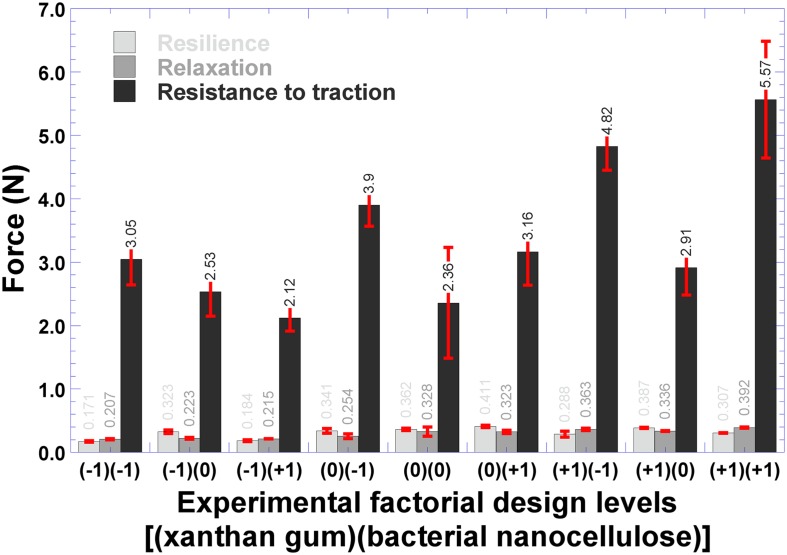
Results gathered from the mechanical resistance tests performed to the plain biofilms evolved from the experimental factorial design.

The experimental factorial design (Table 1) undertaken to define the best biopolysaccharide composition and associated mechanical properties for the biofilm yielded as best composition for the biofilm those of the level (+ 1)(+ 1) [(bacterial nanocellulose)(xanthan gum)] (shaded row in Table 1). Bacterial nanocellulose was chosen as one of the biopolymers to integrate the biopolymeric film due to its interesting characteristics for application in biomedical devices (Petersen and Gatenholm, 2011; Rajwade et al., 2015). Departing from this (optimal) composition leading to the biofilm with the highest values of resistance to traction and relaxation (Figure 5), two biofilms were produced (Table 1) integrating the same factorial level of (bacterial nanocellulose) (xanthan gum) [viz. (+1)(+1)] and human insulin, and integrating or not ionic liquid (CG 1:2). In a review paper by Baker et al. (2012), these researchers claim that it is possible to produce soft hydrogels with ca. 10% (w/w) PVA and rigid hydrogels with ca. 50 to 60% (w/w) PVA. The major purpose of the research effort described herein was to produce a biopolysaccharide film with malleability and plasticity, although with a certain resistance to traction so that it could be utilized for topical applications in the transdermal delivery of insulin. Evaluation of the mechanical properties of the biofilms produced without insulin, according to the factorial design and composition displayed in Table 1, encompassed resistance to traction, relaxation and resilience (Figure 5).

According to the results displayed in Figure 5, the best results produced in terms of resistance to traction, relaxation and resilience were obtained for the levels (+ 1) of bacterial nanocellulose and (+ 1) of xanthan gum. The ability of the biofilm to spring back into shape following deformation caused by straining was an especially important attribute, since its elasticity (i.e., its resilience) is an important characteristic for skin applications. Hence, this set of biopolysaccharide levels was followed for the production of biofilms loaded with human insulin (Table 1). The mechanical properties of the biofilms are mainly related with the biopolymer’s ability to form bonds in polymer chains, leading to resistance to their separation when subject to mechanical forces (Yang, 2012). The plasticizer used (glycerol, in this case) also has an influence on these properties (Bourtoom, 2008).

### Thermal Analysis of the Optimized Biopolymeric Film Integrating Human Insulin and Ionic Liquid, via DSC

**FIGURE 6:**
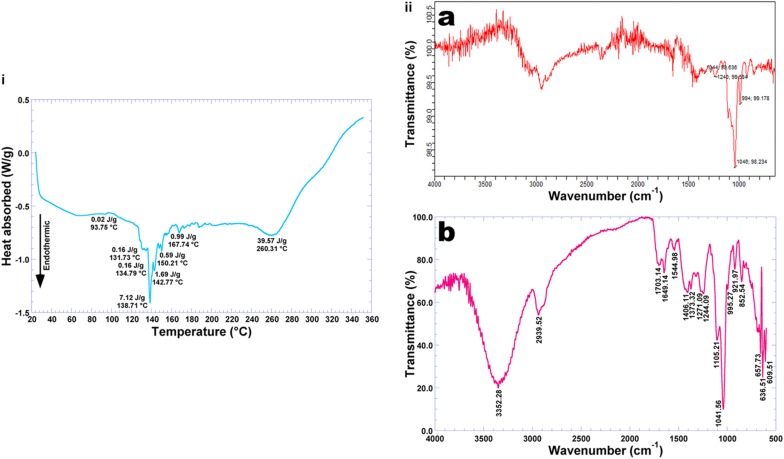
**(i)** Differential scanning calorimetry thermogram of the biofilm integrating choline geranate 1:2 and insulin NOVOLIN^®^ N; **(ii)** FTIR spectra of **(a)** plain insulin NOVOLIN^®^ N and **(b)** biofilm integrating choline geranate 1:2 and insulin NOVOLIN^®^ N.

The results from DSC analysis of a sample of the biofilm integrating human insulin and CG 1:2, recorded under heating mode between 20 and 350°C, are displayed in Figure 6i. The endothermic peak observed at 93.75°C (human insulin moiety, with associated melting enthalpy of 0.020 J/g) may be considered as the first order transition commonly observed in a broad class of hydrated biopolymers such as proteins. By analogy with the work by Dandurand et al. (2014), this endothermic event is attributable to the evaporation of bound water molecules. This endothermic event is associated to orderdisorder transitions, which can be considered as thermal signatures of protein (irreversible) denaturation (Rocha et al., 2017).

The DSC technique of analysis measures the enthalpy of the samples, and may indicate the glass transition temperature and endothermic and/or exothermic events in the samples under scrutiny. The biofilm produced according to the experimental factorial design and integrating HI and CG 1:2, possessed in its composition 1.25% (w/w) PVA with a degree of hydrolysis of 98%, 2.50% (w/w) glycerol, and 0.1% (w/w) methylparaben. The thermogram obtained (Figure 6i) presented one large endothermic event at 138.71°C (with associated melting enthalpy of 7.12 J/g), probably associated with protein carbonization and water loss from the film, and another large endothermic event at 260.31°C (with associated melting enthalpy of 39.57 J/g), probably related to thermal degradation of the carbon moieties of the biopolymeric matrix constituting the film. Since the biofilm integrated human insulin and added water, together with a low amount of ionic liquid, the data obtained from the DSC analysis indicated that the levels utilized did not compromise the stability of the biofilm for the intended use.

### FTIR Analysis of the Optimized Biopolymeric Film Integrating Human Insulin and Ionic Liquid

FTIR spectrophotometry may allow to clarify possible interactions between the loaded protein (human insulin) and the biopolymeric matrix, via analysis of the functional groups present in the different constituents involved in the process. The infrared spectra of **(a)** liquid human insulin (NOVOLIN^®^ N) and **(b)** biofilm integrating human insulin NOVOLIN^®^ N and CG 1:2, are displayed in Figure 6i.

Comparing the spectrum of plain NOVOLIN^®^ N human insulin (Figures 6ii-a, 7i-h) with the spectrum of the biofilm integrating human insulin NOVOLIN^®^ N and CG 1:2, the same characteristic peaks can be observed with only minor variations in peak intensity, viz. at wavenumbers 1244, 1105, 1042, and 995 cm^–1^. This clearly suggests that the chemical aspect of insulin was preserved during integration into the biopolymeric film. According to Gupta et al. (2014), protein molecules may present characteristic energy absorption between wavenumbers 1650 to 1630 cm^–1^ for primary amides, between wavenumbers 1540 to 1520 cm^–1^ for secondary amides and between wavenumbers 1270 to 1230 cm^–1^ for tertiary amides. From inspection of the transmittance spectra depicted in Figures 6ii-a, 7i-h, one can observe peaks within the wavenumber range probably representing primary amides at approximately 1620 cm^–1^ due to the stretch of the carbonyl group (C = O), secondary amides at approximately 1519 cm^–1^ and tertiary amides in the region of 1238 cm^–1^ due to the stretch of the bond C-N. Additionally, the positions of these peaks confirm the protein, such as 1650 cm^–1^ (random coil), and 1613 cm^–1^ (beta-sheet), for primary amide, 1540 cm^–1^ (random coil) and 1520 cm^–1^ (beta-sheet) for secondary amide, and 1270 cm^–1^ (beta-sheet) and 1230 cm^–1^ (random coil) for the tertiary amide (Gupta et al., 2014). The biofilm (Figure 6ii-b) produced a very similar infrared spectrum, with a major peak produced around 3352 cm^–1^ corresponding to OH groups in water, and another major peak at 1649 cm^–1^ (Figure 6ii-b) corresponding to a stretch of the C = O group of primary amides, most likely due to the strong hydrophilic character of primary amides. The stretching vibrations observed in the wavenumber region of 3352 cm^–1^ (Reis et al., 2006), accounting for the large peak observed is most likely accounted for by hydroxyl groups from water molecules present in the film. Characteristic peaks indicative of protein lie between 1390 and 1250 cm^–1^ for C-N bonds, and between 1640 and 1500 cm^–1^ for N-H groupings (Mehta et al., 2013). The infrared spectra displayed in Figure 6ii clearly suggest that the chemical aspect of the protein moiety was preserved during production of the biofilm, allowing to conclude that the insulin protein did not engage in any chemical interactions with the biofilm components, only being carried by the film, which otherwise could have reduced its bioactivity.

### Mechanical Resistance Properties of the Optimized Biopolymeric Film Integrating Human Insulin and Ionic Liquid

The results produced during evaluation of the mechanical resistance of the biofilm integrating insulin and ionic liquid loads, with respect to resistance to traction, relaxation and resilience, are displayed in Figure 8.

**FIGURE 7:**
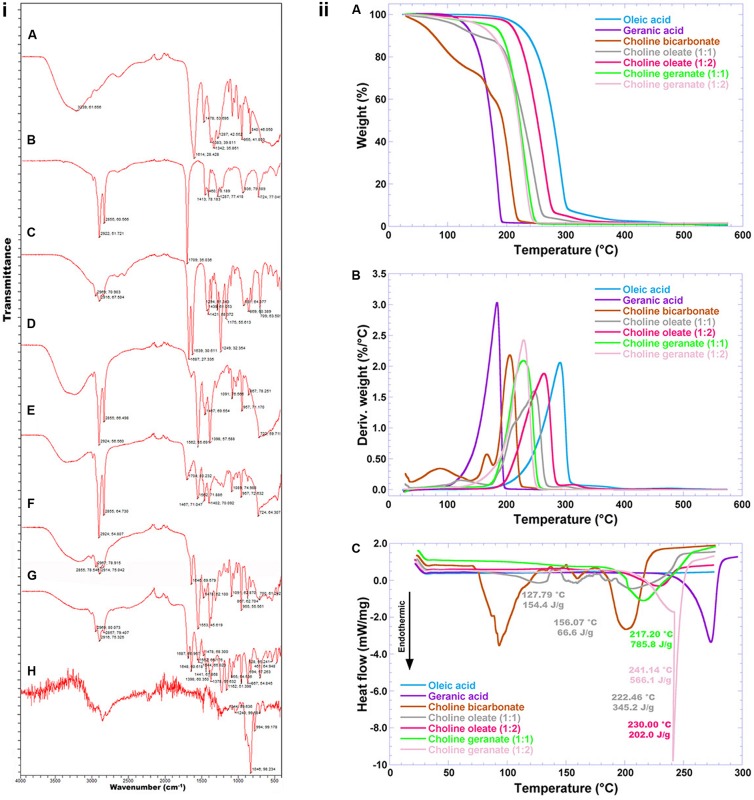
**(i)** FTIR spectra of **(A)** choline bicarbonate, **(B)** oleic acid, **(C)** geranic acid, **(D)** choline oleate 1:1, **(E)** choline oleate 1:2, **(F)** choline geranate 1:1, **(G)** choline geranate 1:2, and **(H)** human insulin (NOVOLIN^®^ N); **(ii)** Thermogravimetric curves **(A)**, 1st derivative of the weight loss curves **(B)**, and differential scanning calorimetry thermograms of plain ionic liquids and their base reactants. The analyses were carried out in a differential scanning microcalorimeter from TA Instruments (model MDSC 2910, New Castle, DE, United States), using high-pressure aluminum pans duly sealed by pressure.

**FIGURE 8:**
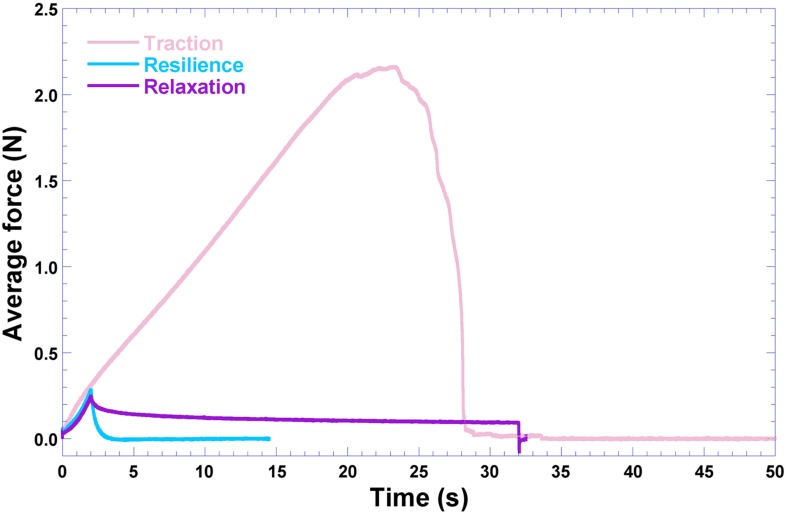
Results gathered from the mechanical resistance tests performed to the biofilm produced with choline geranate 1:2 and insulin NOVOLIN^®^ N, having as base the levels (+1) of bacterial nanocellulose and (+1) of xanthan gum.

**FIGURE 9:**
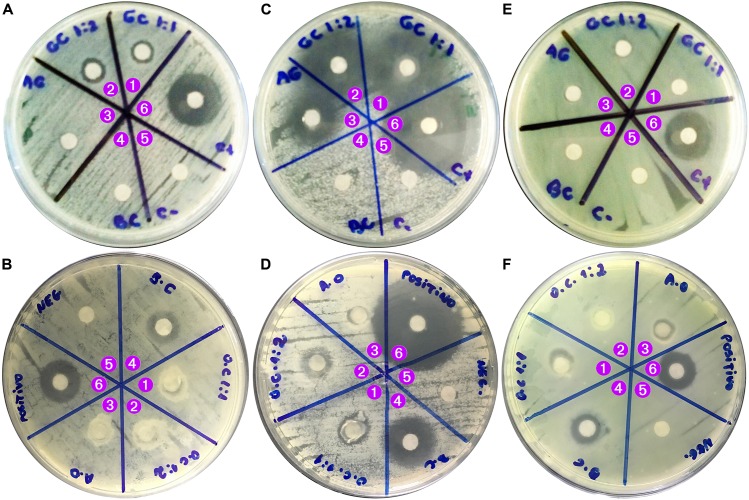
Results obtained from the antimicrobial activity assays performed to the synthesized ionic liquids and their base reactants. **Hexant 1:** sterile filter paper disk impregnated with ionic liquid (1:1; 10 μL choline geranate, 27.1 μL choline oleate); **Hexant 2:** sterile filter paper disk impregnated with deep eutectic solvent (1:2 ionic liquid; 10 μL choline geranate, 20.9 μL choline oleate); **Hexant 3:** sterile filter paper disk plunged into the acid solution (either geranic or oleic acids); **Hexant 4:** sterile filter paper disk plunged into choline bicarbonate solution; **Hexant 5:** sterile filter paper disk plunged into saline solution (0.9%, w/w); **Hexant 6:** sterile filter paper disk impregnated with 10 μL of an antibiotic cocktail solution [penicillin (10000 U.I./mL)/streptomycin (10 mg/mL)]. The impregnated disks were applied directly on the surface of TSA culture medium inoculated via smearing with a suspension of *Escherichia coli* **(A,B)**, *Staphylococcus aureus* **(C,D)** or *Pseudomonas aeruginosa* **(E,F)**. As positive and negative controls, penicillin/streptomycin and saline solution (0.9% NaCl, w/w) were used, respectively.

From inspection of the data displayed in Figures 5, 8, the polysaccharide levels selected produced increased resistance to traction and relaxation of the resulting biofilm. Hence, it may be concluded that the biopolysaccharide levels selected improved the viscoelastic characteristics of deformation and molecular relaxation (viz. return of the biofilm from strained conditions back into equilibrium) (Gong and Hong, 2012). The major goal of the research work undertaken was to develop a biofilm based on biopolysaccharides and impregnated with CG 1:2 (DES) and HI, suitable for application on the skin aiming at the delivery of insulin via transdermal permeation. In our perspective, the biofilm applied directly to the skin should not strongly adhere, allowing release of insulin and easy removal of the spent biofilm from the skin. Thus, resistance to traction, relaxation and resillience were the mechanical resistance parameters evaluated, whereas adhesiveness or bioadhesiveness were not at all considered important in the research work developed. The biofilm is also intended to help in maintaining a suitable microclimate on the skin surface, of utmost importance for the biosynthetic reactions necessary for cellular activities (Boateng et al., 2008). The developed biofilm possesses PVA in its composition, which is a hydrophobic polymer with a low surface free energy (Asadinezhad et al., 2012) that do not favor attraction of the water molecules present in the dispersion containing the polymers. As a consequence, this promotes a non-strong adherence of the biofilm to the skin. The synthesized ionic liquids and their base reactants were screened for any potential antimicrobial activity (Figure 9) via the agar-diffusion method. Sterile filter paper discs (ca. 7.0 mm in diameter) were impregnated by plunging in the ionic liquids: IL (1:1), 27.1 μL; IL (1:2), 20.9 μL. Hexant 1: 1:1; 10 μL CG, 27.1 μL CO; Hexant 2: 1:2; 10 μL CG, 20.9 μL CO); Hexant 3: GA or OA; Hexant 4: CB; Hexant 5: saline solution (0.9%, w/w); Hexant 6: 10 μL penicillin (10000 U.I./mL)/streptomycin (10 mg/mL). The impregnated disks were applied directly on the surface of TSA culture medium innoculated via smearing with a suspension of *Escherichia coli* (Figures 9a,b), *Staphylococcus aureus* (Figures 9c,d) or *Pseudomonas aeruginosa* (Figures 9e,f). As positive and negative controls, penicillin/streptomycin and saline solution (0.9% NaCl, w/w) were used, respectively. As can be observed from inspection of Figure 9, CG 1:1 and CG 1:2 exhibited positive antimicrobial activity against *Escherichia coli* (Figure 9a, hexants 1 and 2) and *Staphylococcus aureus* (Figure 9c, hexants 1 and 2), but had no effect whatsoever upon *Pseudomonas aeruginosa* (Figure 9e, hexants 1 and 2). On the contrary, CO 1:1 and CO 1:2 had no effect whatsoever upon *Escherichia coli* (Figure 9b, hexants 1 and 2), but both CO ionic liquids exhibited only a mild antimicrobial activity against *Staphylococcus aureus* (Figure 9d, hexants 1 and 2). On *Pseudomonas aeruginosa*, only CO 1:1 showed a positive mild antimicrobial activity (Figure 9f, hexants 1 and 2).

### Cytotoxicity Potential of Plain Biofilm and Biofilm Integrating Choline Geranate and Human Insulin

**FIGURE 10:**
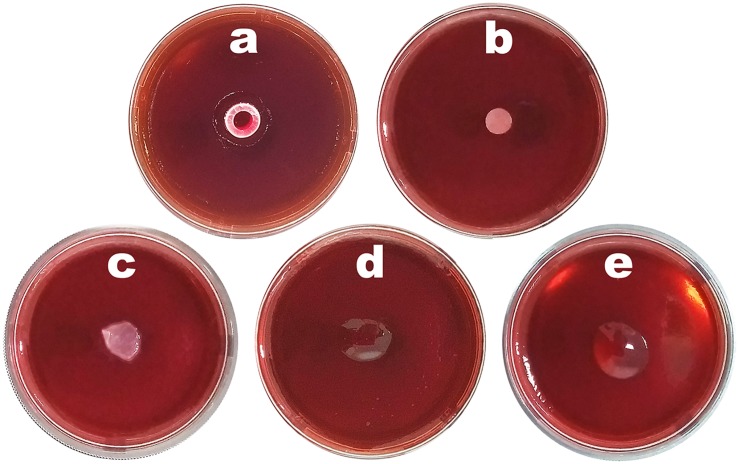
Results from the cytotoxicity analysis of plain bacterial nanocellulose membrane, plain biofilm without insulin NOVOLIN^®^ N and without choline geranate 1:2, and biofilm integrating both insulin NOVOLIN^®^ N and choline geranate 1:2, via the agar disk-diffusion methodology using the cell lineage HaCaT: **(a)** positive control, **(b)** negative control, **(c)** bacterial nanocellulose, **(d)** plain biofilm, **(e)** biofilm integrating insulin NOVOLIN^®^ N and choline geranate 1:2.

The cytotoxicity potential of bacterial nanocellulose, of the plain biofilm (without human insulin and without ionic liquid) and of the biofilm integrating both ionic liquid and human insulin, together with a positive and a negative control, were evaluated via the agar disk-diffusion methodology using the cell lineage HaCaT (Figure 10).

The results obtained showed that there was no cell death whatsoever caused by contact with either the nanocellulose biomembrane (Figure 10c) or the biofilms produced without HI and IL (Figure 10d) or the biofilm integrating both IL and HI (Figure 10e), as apparent from the absence of any clearer halos around the material samples. The readings of the inoculated plaques were made macroscopically, where the absence of cytotoxicity was observed by the lack of formation of a clear halo around the material (which would otherwise correspond to dead cells), and microscopically, for any morphological changes of the cells surrounding the sample. Hence, the biofilm produced and integrating both IL and HI was not cytotoxic and can therefore be considered safe for application on the skin.

### Structural Microanalysis of the Biofilm Integrating Choline Geranate and Human Insulin, via DESEM Analyses

**FIGURE 11:**
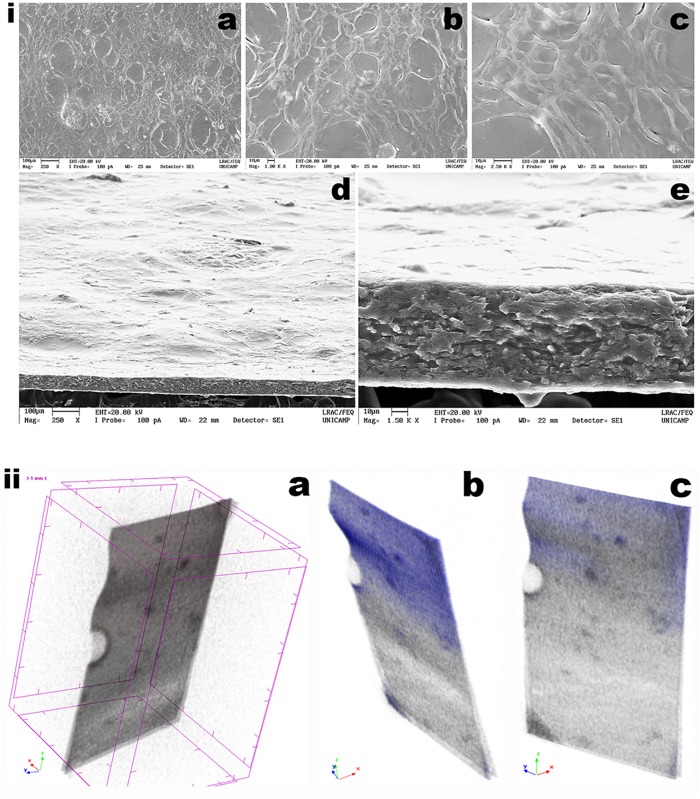
**(i)** DESEM photomicrographs of the biofilm loaded with insulin NOVOLIN^®^ N and choline geranate 1:2, at several magnifications (biofilm surface, **a:** ×250, **b:** ×1000, **c:** ×2500; biofilm fracture cross-section, **d:** ×250, **e:** ×1500), gathered using the electron back-scattered diffraction (EBSD) technique; **(ii)** Images obtained by tomographic analyses via X-ray transmission of the biofilm loaded with insulin NOVOLIN^®^ N and choline geranate 1:2, being **(a,b)** slant profile images of the biofilm, **(c)** image perpendicular to the surface of the biofilm. Three-dimensional image slices were gathered using an operating voltage set at 34 kV and electric current with 529 μA.

The DESEM images of both the surface and fracture cross-section of the biofilm loaded with HI and CG 1:1 allows observing a smooth surface with generalized absence of cracks (Figure 11).

Probably due to the process involved in preparation of the samples prior to sputter coating with colloidal Au, fixation of the films in the carbon supports by stretching them might have produced the microscopic cracks observable in the photomicrographs **a**, **b,** and **c** in Figure 11i, especially for higher magnifications. Nevertheless, from the results gathered in the scanning electron microscopy analyses performed to the optimized biofilm, a uniform morphology could be observed, at three high magnifications [viz. ×250 (Figure 11i-a), ×1000 (Figure 11i-b), ×2500 (Figure 11i-c)]. The fractured cross-section of the biofilm exhibit a highly homogeneous matrix of the biopolymeric structure, which facilitate insulin release upon contact of the biofilm with (moist) skin. It can be observed that the topography in the film biostructure displayed in Figures 11i-d,e is highly homogeneous.

### XRT Analysis of the Biofilm Integrating Choline Geranate and Human Insulin

The optimized biofilm developed may be considered a natural polymer composite exhibiting a very special porous microstructure that enables the film to possess outstanding mechanical properties (as concluded from inspection and analysis of the results depicted in Figure 8). From the tomographic analyses via X-ray transmission performed to a rectangular section of the biofilm loaded with HI and CG (Figure 11ii), a homogeneous surface can be observed.

Due to absorbing more radiation, because of its higher atomic density, the biopolysaccharide matrix network (in gray and blue in Figure 11i) in greater evidence, whereas void spaces appear pinpointed in black throughout the reconstituted three-dimensional image of the film (Figure 11i). These results are in close agreement with those obtained by FTIR (Figure 6ii-b) which indicated that insulin probably did not engage in any chemical bonding with the biopolymeric matrix. This is clearly an important and positive data, since by not engaging in any chemical bonding with the biopolymeric matrix, insulin becomes readily available and maintains its bioactivity. A comparative porosity analysis of the biofilm integrating HI and CG 1:2, resulting from 2D and 3D morphological analyze in Supplementary Material.

### Ionic Liquid-Assisted Transdermal Permeation of Human Insulin

**FIGURE 12:**
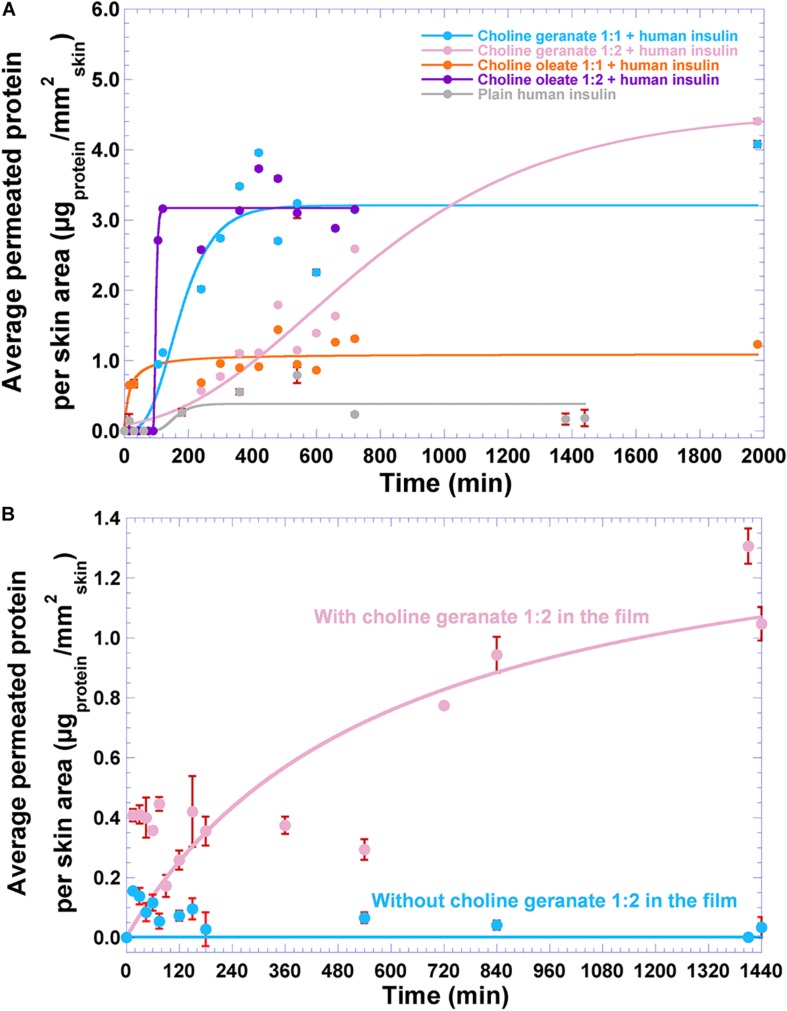
Results gathered from transdermal permeation studies, as **(A)** average permeated protein (insulin NOVOLIN^®^ N) per skin area using plain ionic liquids, and **(B)** average permeated protein (insulin NOVOLIN^®^ N) per skin area from the biofilm integrating insulin and choline geranate 1:2, during a 24 h-timeframe assay. Values are the means of three experiments (*n* = 3), with associated standard deviations. Solid lines represent non-linear fittings performed to the data, viz. hyperbolic for the transdermal permeation data using plain choline oleate 1:1 and for the transdermal permeation data using the biofilm integrating insulin and choline geranate 1:2, and Gompertz function for all other permeation assays.

The results obtained from the transdermal permeation of insulin aided by plain ionic liquids are displayed in Figure 12a.

As can be observed from inspection of Figure 12a, the presence of ionic liquid led to increased average permeated protein per skin area, as opposite to the permeation trial with plain insulin (gray curve in Figure 12a). Performing non-linear (hyperbolic, *y* = (*m*_1_*x*)/(*m*_2_ + *x*)) fittings to the results obtained from transdermal permeation of protein facilitated by plain CO 1:1 (Table 2 and Figure 12a, orange curve) and to the results obtained from transdermal permeation of protein from the biofilm integrating insulin and CG 1:2 (Table 2 and Figure 12b, light pink curve), one obtained the maximum average protein permeated per skin area (parameter m_1_, Table 2) and the time required to achieve half of the maximum average protein permeated per skin area (parameter m_2_, Table 2), with excellent correlation coefficients. Regarding the results from transdermal permeation using the remainder plain ionic liquids, and also the results arising from transdermal permeation of plain insulin from the biofilm without choline geranate 1:2, the sigmoidal trends of the protein permeation data was best described by a non-linear fit of the Gompertz function (*y* = *m*_1_*e*^−*m*_2_*e*^−*m*_3_*x*^^, Figures 12a,b and Table 2), allowing to obtain the value of the asymptote (m_1_, i.e., the maximum attainable permeated protein per skin area, Table 2), the displacement of the data trend along the timeframe studied (viz. m_2_, Table 2) and the data growth rate (viz. m_3_, Table 2), with good correlation coefficients. The Gompertz function is a sigmoid function, where growth is slowest at both start (where protein concentration was initially high, so skin uptake was slow) and end (due to slowing of protein permeation as saturation was reached in the skin) of a given time period.

**TABLE 2:** Results obtained from the non-linear fittings performed to the average permeated protein per skin area ($\mu g_{protein}/mm_{skin}^{2}$) as a function of permeation time, for both plain mixtures of human insulin and ionic liquids and for the biofilms integrating ionic liquid (choline geranate 1:2) and loaded (or not) with human insulin.

Sample	Hyperbolic function: *y* = (*m*_1_*x*)/(*m*_2_ + *x*)		Gompertz function: *y* = *m*_1_*e*^−*m*_2_*e*^−*m*_3_*x*^^			*r*
	m_1_	m_2_	m_1_	m_2_	m_3_	
Plain choline geranate 1:1 + human insulin	—–	—–	3.2065	7.2967	0.01372	0.95855
Plain choline geranate 1:2 + human insulin	—–	—–	4.5423	4.0200	0.002396	0.97723
Plain choline oleate 1:1 + human insulin	1.0934	15.849	—–	—–	—–	0.83923
Plain choline oleate 1:2 + human insulin	—–	—–	3.1699	4.1891 × 10^9^	0.2286	0.98815
Plain human insulin	—–	—–	0.3865	67.6410	0.02888	0.67167
Biofilm with choline geranate 1:2 and human insulin	1.5138	596.48	—–	—–	—–	0.75531
Plain biofilm with human insulin	—–	—–	0.001654	−0.8695	1.6417	1.00000

As can be observed from inspection of Figure 12 and the data in Table 2, the biofilm integrating insulin and CG 1:2 [the plain ionic liquid that performed well in terms of lack of cytotoxicity (Figures 3i,ii-a), genotoxicity (Figures 3iii-a,b), and permeation enhancement (Figure 12a, light pink curve)] allowed an average permeated protein per skin area of 1.05 μg_*protein*_/mm^2^_*skin*_.

## Conclusion

In the research effort just described, synthesis of ILs and their DES was pursued aiming at their use as potential enhancers of insulin transdermal permeation, followed by their physicochemical and biological characterization. The CG 1:2 DES was chosen to be incorporated in the biofilm due to its non-cytotoxic and non-genotoxic characteristics, associated with a superior promotion of insulin transdermal permeation. The biopolymeric film developed displayed a good characteristic in relation to release of the active insulin moiety, verified through transdermal permeation studies. The biofilm loaded with CG 1:2 and HI was found to be suitable for biopharmaceutical applications, namely in the delivery of insulin via transdermal permeation, a conclusion fully supported by the results gathered from the physicochemical and biological characterization that was entailed.

## Data Availability Statement

The datasets generated for this study are available on request to the corresponding author.

## Author Contributions

LJ, LH, ES, WC, and FM were responsible for carrying out various laboratory tests. GS was responsible for carrying ^1^H NMR analyses. JP collaborated with the interpretation of results. JO was responsible for the tomographic analyses via X-ray transmission (XRT). MT and MV collaborated with the interpretation of results obtained and critical reading of the manuscript. VB was responsible for the conception and drafting the manuscript.

## Conflict of Interest

The authors declare that the research was conducted in the absence of any commercial or financial relationships that could be construed as a potential conflict of interest.
