# Supplementary material for: Non-invasive Transdermal Delivery of Human Insulin Using Ionic Liquids: In vitro Studies
Source: Front Pharmacol. 2020 Apr 23;11:243. doi: 10.3389/fphar.2020.00243 (PMC7191306; doi:10.3389/fphar.2020.00243)
Supplement: Supplementary file 1 [file Data_Sheet_1.docx]

**Supplementary Material**

1. Results obtained from the tomographic analyses via X-ray transmission performed to the biofilm integrating ionic liquid (CG1:2) and human insulin.

| **Parameter** | **Biofilm integrating choline geranate 1:2 and 1.500 mL human insulin (NOVOLIN®)** | |
| --- | --- | --- |
|  | **Bi-dimensional (2D) morphological analysis** | **Three-dimensional (3D) morphological analysis** |
| Number of layers | ---------- | 101.000 |
| Pixel size (µm) | 9.80 | 9.7610 |
| Total VOI (volume of interest), TV (mm^3^) | 0.07991 | 0.07860 |
| Object volume, Obj.V (mm^3^) | 0.05263 | 0.05043 |
| Percent object volume, Obj.V/TV (%) | 65.8696 | 64.1641 |
| Total VOI surface, TS (mm^2^) | 4.6433 | 4.19547 |
| Object surface, Obj.S (mm^2^) | 6.0601 | 5.36646 |
| Intersection surface, i.S (mm^2^) | 1.6320 | 1.45215 |
| Object surface / volume ratio, Obj.S/Obj.V (mm^-1^) | 115.1370 | 106.4041 |
| Crossectional thickness, Cs.Th (mm) | 0.02341 | ---------- |
| Object surface density, Obj.S/TV (mm^-1^) | ---------- | 68.2733 |
| Degree of anisotropy, DA | ---------- | 6.56898 (0.84777) |
| Eigenvalue 1 | ---------- | 0.10124 |
| Eigenvalue 2 | ---------- | 0.15189 |
| Eigenvalue 3 | ---------- | 0.66505 |
| Number of closed pores, Po.N(cl) | ---------- | 63.0000 |
| Volume of closed pores, Po.V(cl) (mm^3^) | ---------- | 0.00006 |
| Surface of closed pores, Po.S(cl) (mm^2^) | ---------- | 0.03219 |
| Closed porosity (percent), Po(cl) (%) | 0.25474 | 0.11783 |
| Mean fragmentation index, Fr.I (mm^-1^) | -9.24357 | -12.5258 |
| Mean fractal dimension, FD | 1.26605 | 2.18725 |
| Volume of open pore space, Po.V(op) (mm^3^) | ---------- | 0.02811 |
| Open porosity (percent), Po(op) (%) | ---------- | 35.76013 |
| Total volume of pore space, Po.V(tot) (mm^3^) | ---------- | 0.02817 |
| Total porosity (percent), Po(tot) (%) | ---------- | 35.83582 |
| Euler number, Eu.N | ---------- | -426.0000 |
| Connectivity, Conn | ---------- | 546.0000 |
| Connectivity density, Conn.Dn (mm^-3^) | ---------- | 6946.337 |

Anisotropy is the property of being directionally dependent, as opposed to isotropy, which implies identical properties in all directions. The degree of anisotropy, calculated as $\text{Degree} \text{of} \text{anisotropy} \text{=} \text{1-}\left(\frac{\text{Eigenvalue}_{\text{min}}}{\text{Eigenvalue}_{\text{max}}}\right)$, is 0 for total isotropy and 1 for total anisotropy. Hence, as can be seen from inspection of the data in Supplementary Material 1, the degree of anisotropy is 0.84777 for the biofilm loaded with HI and CG 1:2, a value that is more close to anisotropy than to isotropy in the aforementioned scale. Being the biofilm loaded with insulin an engineered biomaterial with a woven structure, this result is easily understandable under the light of the DESEM images displayed in Figure 11i. When analyzing the structure of the biofilm loaded with HI and CG 1:2, one finds that it has an open porosity of 35.76% and an almost equal value of total porosity. Additionally, the mean fragmentation index gives an index of connectivity and calculates an index of relative convexity or concavity of the total object surface, on the principle that concavity indicates connectivity (and the presence of “nodes”), and convexity indicates isolated disconnected structures (struts). Lower fragmentation indexes mean better connected lattices while higher fragmentation indexes mean a more disconnected structure. Abundant enclosed cavities and concave surfaces can push the fragmentation index to (large) negative values, which was in fact determined in the analyses performed. These results are quite important since due to the fact that insulin was homogeneously dispersed throughout the biopolymeric structure of the biofilm during the polymerization process, the biofilm can be applied with either of its surfaces towards the skin.
